# Minimally Invasive Endoscopic Surgery in Patients With Previous Cardiac or Thoracic Surgery

**DOI:** 10.1016/j.jaccas.2026.107738

**Published:** 2026-04-03

**Authors:** De Qing F.N. Görtzen, Fleur Sampon, Ismail Cenik, Bart M.J.A. Koene, Pim A.L. Tonino, Joost F.J. Ter Woorst, Ferdi Akca

**Affiliations:** a Department of Cardiothoracic Surgery, Catharina Hospital Eindhoven, Eindhoven, the Netherlands; b Department of Interventional Cardiology, Catharina Hospital Eindhoven, Eindhoven, the Netherlands; c Department of Biomedical Engineering, Technical University Eindhoven, Eindhoven, the Netherlands

**Keywords:** coronary angiography, coronary artery bypass, myocardial infarction, myocardial ischemia, myocardial revascularization, percutaneous coronary intervention, postoperative, stenosis, thoracotomy

## Abstract

**Background:**

Minimally invasive, sternal-sparing techniques offer an alternative to conventional repeat sternotomy in patients with prior cardiothoracic surgery, aiming to achieve durable coronary revascularization while minimizing procedural risk.

**Case Summary:**

Between 2022 and 2025, six patients underwent redo minimally invasive endoscopic-assisted coronary artery bypass grafting (Endo-CAB) with endoscopically harvested graft conduits, and a beating-heart anastomosis on the left anterior descending artery was made through a mini-thoracotomy. This case series presents patients with diverse prior cardiac and thoracic surgical histories. Although percutaneous coronary intervention was considered because of the increased operative risk, a minimally invasive surgical approach was preferred given unfavorable coronary anatomy and advanced atherosclerosis. All patients had an uneventful postoperative recovery.

**Discussion:**

Redo Endo-CAB avoids repeat sternotomy, reduces surgical trauma, and facilitates a safe internal mammary artery harvest. This approach combines the durability of surgical revascularization with reduced perioperative morbidity in selected high-risk patients.

**Take-Home Message:**

Redo Endo-CAB is a viable and promising revascularization strategy for selected patients with prior cardiothoracic surgery when performed at experienced centers.

## Visual Summary

**Figure:**
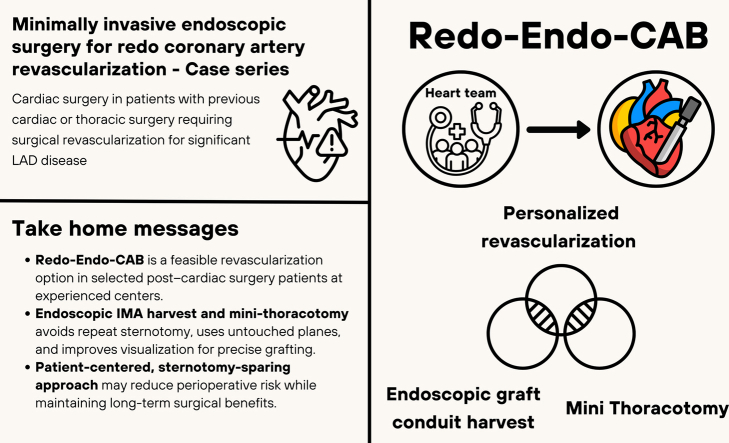

Cardiac surgery in patients with previous thoracic or cardiac surgery is technically demanding and requires meticulous planning. These patients often present with advanced age, complex cardiac pathology, increased comorbidities, and intrathoracic adhesions, which complicate surgical dissection and increase the risk of coagulopathy and intraoperative mortality. Furthermore, redo surgery carries a heightened risk of injury to major intrathoracic vessels, patent coronary grafts, cardiac chambers, and lungs during sternal entry and dissection.^1^ Consequently, patients presenting with recurrent angina often undergo redo percutaneous coronary intervention (PCI) because of lower periprocedural risk compared with redo surgery, despite redo surgery offering superior long-term freedom from repeat intervention.^2^

**Take-Home Messages**

• Redo Endo-CAB is a promising and viable revascularization option for selected patients who have undergone previous cardiothoracic surgery in experienced centers.
• Endoscopic internal mammary artery harvesting with a mini-thoracotomy in redo Endo-CAB avoids repeat sternotomy by operating in untouched anatomical planes, enhances visualization, and allows for more precise revascularization.
• Providing a patient-oriented personal approach and avoiding repeat sternotomy, this approach may significantly reduce perioperative risk while preserving the long-term benefits of surgical coronary revascularization.

Minimizing surgical trauma is essential in considering redo cardiac surgery as a viable option. Recent advances in minimally invasive off-pump surgery, endoscopic conduit harvesting (internal mammary or radial artery), and grafting through a mini-thoracotomy have facilitated this strategy.^3^^,^^4^ A mini-thoracotomy provides a sternal-sparing access to the coronary targets and often avoids extensive mediastinal dissection after prior sternotomy, enabling its use in selected high-risk patients. This approach is associated with reduced postoperative pain, fewer sternal wound complications, and faster recovery.^5^

The use of minimally invasive, sternal-sparing techniques in combination with endoscopic graft conduit harvesting in patients with previous thoracic or cardiac surgery offers a compelling alternative to conventional repeat sternotomy. A sternal-sparing approach aims to achieve durable coronary revascularization while minimizing the procedural risks inherent to redo cardiac surgery. The present case series explores the feasibility and outcomes of such a strategy in patients undergoing redo surgical coronary revascularization.

## Case Series

Between 2022 and 2025, six patients underwent minimally invasive redo cardiac surgery for surgical revascularization of the left anterior descending artery (LAD) at our institution and were included in this case series. All patients presented with new or recurrent angina and were evaluated by the referring cardiologists. Coronary angiography demonstrated significant coronary artery disease, and the heart team discussed all cases. Although percutaneous intervention was considered given high operative risk, advanced atherosclerosis and technical complexity favored surgical revascularization. The preoperative assessment included coronary angiography with fractional flow reserve measurements, chest radiography, electrocardiography, transthoracic echocardiography, laboratory testing, bilateral blood pressure measurements, and computed tomography (CT) to evaluate graft location, mammary artery patency, and accessibility of the ascending aorta and femoral vessels.

The patients in this case series had diverse prior cardiac and thoracic surgical histories, with extensive adhesions in the thoracic cavity (Table 1). Two patients had undergone thoracic surgery (pleurectomy via posterolateral thoracotomy; pectus excavatum repair, including sternal turnover). The remaining patients had prior cardiac procedures, including combined coronary artery bypass grafting (CABG) and aortic valve replacement (AVR), endoscopic-assisted coronary artery bypass grafting (Endo-CAB), isolated CABG, and repeat AVR.

**Table 1:** Characteristics of the 6 Included Cases

Case	Previous Thoracic/Cardiac Surgery	Lesion on Preoperative CAG	Heart Team Decision	Reoperation Graft Formula
1	2 pectus excavatum repairs at the ages of 6 and 12 (caudal sternal turnover)	1-vessel disease, LAD (FFR: 0.73)	Endo-CAB: single graft	Redo Endo-CAB: SVG-LAD
2	Posterolateral thoracotomy left sided pleurectomy	1-vessel disease, LAD (FFR: 0.71)	Endo-CAB: single graft	Redo Endo-CAB: RIMA-LAD
3	AVR + CABG: Ao-SVG-IM	3-vessel disease, occluded Ao-SVG-IM and RCA, LAD significant stenosis	Endo-CAB: single graft	Redo Endo-CAB: LIMA-LAD
4	Mechanical AVR, redo AVR endocarditis	1-vessel disease, LAD (FFR: 0.72)	Endo-CAB: single graft	Redo Endo-CAB: LIMA-LAD
5	CABG: LIMA-LAD, Ao-SVG-OM	2-vessel disease, left main stenosis, occluded LIMA-LAD, patent SVG, ectatic coronaries	Endo-CAB: single graft	Redo Endo-CAB: RIMA-radial-LAD
6	Endo-CAB: LIMA-LAD, radial-Y–IM	2-vessel disease, patent radial artery graft, tapering of LIMA-LAD	Endo-CAB: single graft	Redo Endo-CAB: LIMA-radial-LAD

Ao = aorta; AVR = aortic valve replacement; CABG = coronary artery bypass grafting; CAG = coronary angiography; Endo-CAB = endoscopic-assisted coronary artery bypass grafting; FFR = fractional flow reserve; IM = intermediate branch; LAD = left anterior descending artery; LIMA = left internal mammary artery; OM = obtuse marginal branch; RCA = right coronary artery; RIMA = right internal mammary artery; SVG = saphenous vein graft.

## Technique Description

The operative strategy was to perform a minimally invasive, sternal-sparing approach, with the possibility to convert to a median sternotomy if the exposure or safety could not be ensured. Access for graft conduit harvesting was obtained using thoracoscopic ports as routinely performed by our center during Endo-CAB procedures, as previously described.^5^ Three small incisions were made on the left side of the thorax; 5-mm endoscopic ports were placed, 1 at the third intercostal space at the anterior axillary line, and 2 more at the second and fourth intercostal spaces, positioned more anteriorly; and a capnothorax was created. A 5-mm, 0°, 2-dimensional camera (Karl Storz GmbH) and standard long-shafted video-assisted thoracic surgery instruments were introduced. Initial thoracoscopic inspection focused on the extent of pleural adhesions, which were carefully dissected using the Ligasure Maryland device (Medtronic). The pericardium was subsequently opened anterior to the phrenic nerve to identify the coronary targets, assess prior grafts, and determine the optimal grafting strategy. In patients with previous coronary bypass surgery using the left internal mammary artery (LIMA), an endoscopically harvested right internal mammary artery (RIMA), radial artery, or saphenous vein graft (SVG) was used. Our technique allows for endoscopic harvest of the RIMA crossing the sternal midline from the left side of the thorax as well.

Minimally invasive conduit harvesting was performed after confirming the feasibility of the minimally invasive approach. Systemic heparinization was then administered, followed by creation of a mini-thoracotomy using the needle method as described by Ohtsuka et al^6^ (third to fifth intercostal space, anatomy-dependent). During the procedure, rib retraction was used to achieve optimal exposure when making the anastomosis and reducing the size of the incision. The graft configuration (in situ internal mammary artery [IMA] grafts, composite Y/T grafts) was tailored to conduit availability and target vessel location. The use of pericardial traction sutures is limited as cardiac rotation is usually restricted by adhesions, and the pericardium is opened as little as possible just to expose the target coronary artery. Furthermore, an epicardial stabilizer (Octopus Nuvo, Medtronic) was used.^7^

**Figure 1:**
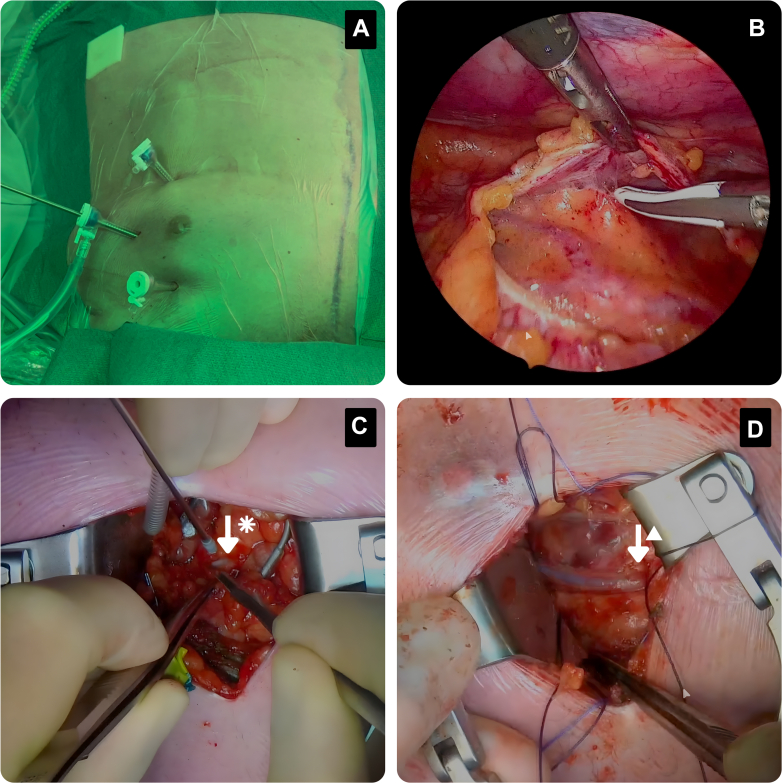
Overview of the Surgical Technique of the Endo-CAB Procedure (A) Surgical setup during the redo Endo-CAB procedure. The previous sternotomy incision is marked with a blue line, and three 5-mm trocars are placed at the left thorax. (B) Endoscopic adhesiolysis of the pericardium to identify the coronary targets. (C) Surgical view of the LAD (arrow with asterisk) stabilized with the epicardial stabilizer before the coronary anastomosis. (D) Final result with a new bypass graft (arrow with triangle) placed on the LAD. Endo-CAB = endoscopic-assisted coronary artery bypass grafting; LAD = left anterior descending artery.

After completion of revascularization, protamine was administered, a drain was placed in the left pleural cavity, and intercostal local anesthesia was administered. The incisions were then closed in standard fashion (Figure 1).

## Patient 1

**Figure 2:**
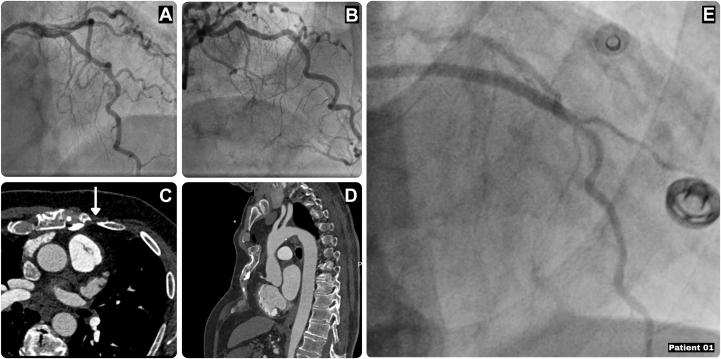
Characteristics of Patient 1 (A and B) Preoperative angiography. (C) Preoperative computed tomography with the bony overgrowth. The arrow denotes the place where the LIMA should be expected. (D) Preoperative computed tomography showing the sternum after 2 repairs for pectus excavatum. (E) Postoperative result with the SVG-LAD graft in situ. LAD = left anterior descending artery; LIMA = left internal mammary artery; SVG = saphenous vein graft.

A 73-year-old man presented with new-onset stable angina and exertional dyspnea, CCS (Canadian Cardiovascular Society) classII. His history included childhood extensive pectus excavatum repairs with caudal sternal turnover, diabetes mellitus, hypertension, varices, and prior smoking. Coronary angiography demonstrated a functionally significant proximal LAD lesion (fractional flow reserve: 0.73), with nonsignificant disease in the circumflex artery and right coronary artery (RCA). CT imaging showed a patent proximal LIMA, with severe bony overgrowth over its medial and distal part (Figure 2). Transthoracic echocardiography revealed normal ventricular and valvular function. A redo Endo-CAB procedure was planned given the previous caudal sternal turnover to reduce sternal trauma (EuroSCORE II: 1.44). During surgery, the thoracoscopic inspection confirmed that LIMA harvesting was not feasible because of bony overgrowth. Pump-assisted venous revascularization using femoral extracorporeal circulation was therefore performed, allowing for safe decompression of the pulmonary artery and optimal exposure of the ascending aorta for the proximal anastomoses. The proximal anastomosis was performed with beating-heart techniques using retraction sutures at the right-sided pericardium next to the aorta, the epicardial stabilizer on the pulmonary artery for additional exposure, and a Lambert-Kay aorta clamp on the ascending aorta. The postoperative course was uncomplicated, with discharge on postoperative day 3. Postoperative coronary angiography demonstrated a patent aorta-SVG-LAD graft.

## Patient 2

**Figure 3:**
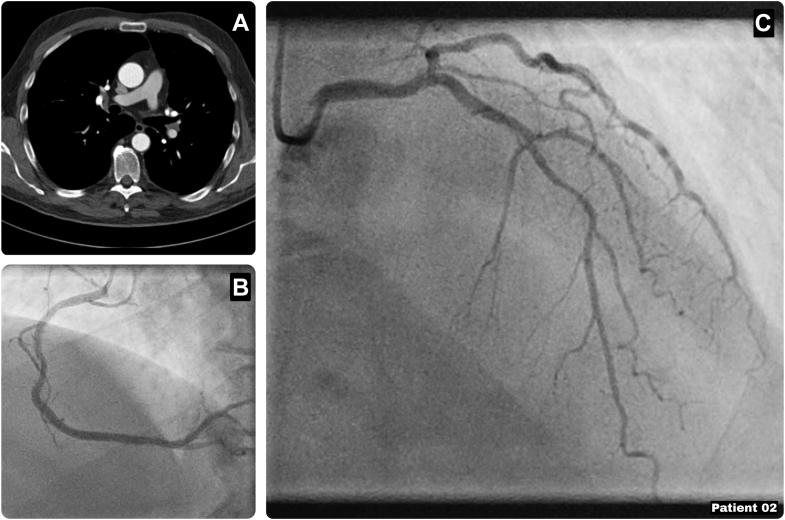
Characteristics of Patient 2 (A) Preoperative computed tomography demonstrating patent bilateral internal mammary arteries without aortic calcifications. (B and C) Coronary angiography showing a significant LAD stenosis (fractional flow reserve: 0.71), with nonsignificant lesions in the diagonal and circumflex artery and a patent RCA stent. LAD = left anterior descending artery; RCA = right coronary artery.

A 67-year-old man was referred for evaluation of palpitations without anginal symptoms and was found to have a prognostically significant LAD lesion. His history included prior non–ST-segment elevation myocardial infarction treated with a PCI of the right coronary artery (RCA), paroxysmal atrial fibrillation, and a left-sided pleurectomy through a posterolateral thoracotomy. Coronary angiography showed a significant LAD stenosis (fractional flow reserve: 0.71), with nonsignificant lesions in the diagonal and circumflex artery and a patent RCA stent. CT imaging demonstrated patent bilateral internal mammary arteries without aortic calcifications, and echocardiography showed normal ventricular and valvular function (Figure 3). After heart team discussion, a redo Endo-CAB using the RIMA was planned owing to anticipated left pleural adhesions (EuroSCORE II: 0.88). During surgery, we entered the right side of the chest, as this was an untouched operative field, and the RIMA was harvested. Subsequently, we crossed the midline endoscopically to perform limited dissection of the left-sided adhesions covering the LAD. Off-pump RIMA-LAD grafting was successfully performed via a left mini-thoracotomy. The postoperative course was uneventful, and the patient was discharged on postoperative day 6 and remained free of anginal symptoms at follow-up.

## Patient 3

**Figure 4:**
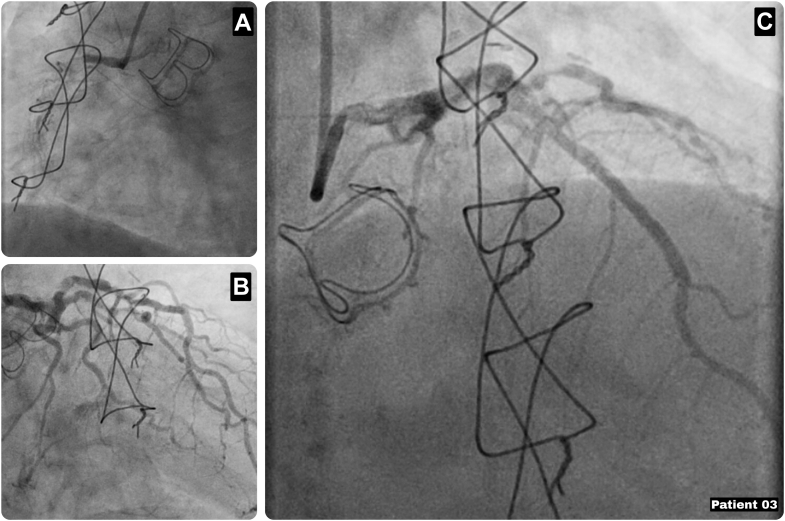
Characteristics of Patient 3 (A) Coronary angiography showing the occluded RCA with the sternal wires and aortic valve prosthesis in situ. (B and C) Coronary angiography showing the significant proximal LAD stenosis. LAD = left anterior descending artery; RCA = right coronary artery.

A 62-year-old man presented with progressive angina (CCS class III) and a complex cardiac history, including prior bioprosthetic AVR for infective endocarditis with concomitant CABG with an SVG to the intermediate branch, postoperative aborted inferoposterior myocardial infarction, cerebrovascular accident, peripheral vascular disease, cardiac asthma, and chronic anemia due to gastrointestinal angiodysplasias, along with hypertension, hypercholesterolemia, obesity (body mass index: 32), prior smoking, and previous heavy alcohol use. Coronary angiography revealed diffuse 3-vessel disease, including occlusion of the venous graft to the intermediate branch, and a significant proximal LAD lesion. Revascularization of the intermediate branch or RCA was not pursued owing to poor distal target quality (Figure 4). Echocardiography showed low-normal left ventricular function and a normally functioning aortic bioprosthesis. Given the unfavorable anatomy for PCI, a redo Endo-CAB was planned as a bailout strategy (EuroSCORE II: 4.06). After thoracoscopic pleural adhesiolysis, the LIMA was harvested endoscopically and anastomosed off-pump to the LAD. The postoperative course was uneventful, with discharge on postoperative day 4, and the patient experienced complete resolution of anginal symptoms at follow-up.

## Patient 4

**Figure 5:**
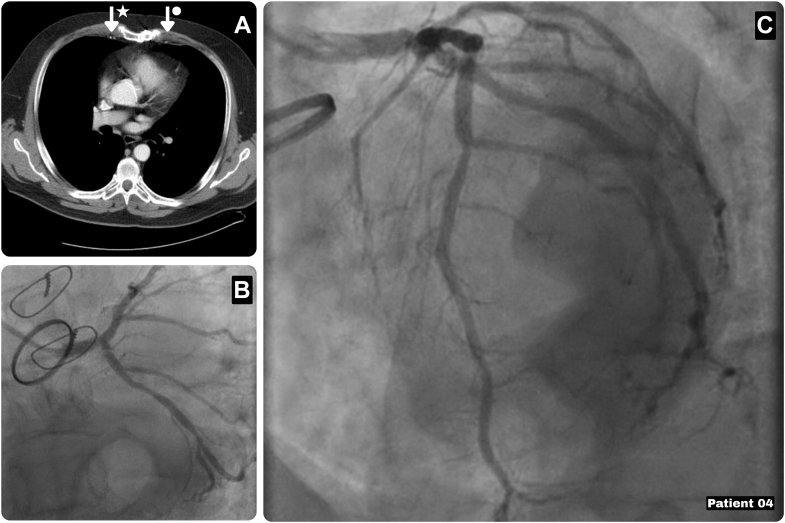
Characteristics of Patient 4 (A) Preoperative computed tomography showing the LIMA (arrow with dot) and RIMA (arrow with star). (B and C) Preoperative coronary angiography with a mechanical aortic valve in situ and a calcified left main coronary artery and a functionally significant LAD lesion (fractional flow reserve: 0.72). LAD = left anterior descending artery; LIMA = left internal mammary artery; RIMA = right internal mammary artery.

A 71-year-old man presented with severe angina (CCS class III-IV) and recurrent nonsustained ventricular tachycardia in the context of 2 prior aortic valve replacements for aortic regurgitation and prosthetic valve endocarditis. His medical history included hypertension, hypercholesterolemia, obesity, and first-degree atrioventricular block and premature ventricular complexes. Coronary angiography demonstrated a calcified left main coronary artery and a functionally significant LAD lesion (fractional flow reserve: 0.72), while the circumflex artery was not hemodynamically significant, and echocardiography showed preserved ventricular function and a normally functioning mechanical aortic valve (Figure 5). Based on preoperative CT confirming LIMA accessibility, a re-redo Endo-CAB was performed (EuroSCORE II: 3.73). Intraoperatively, limited pleural and extensive pericardial adhesions were released, allowing successful LIMA-LAD grafting through a left mini-thoracotomy. Postoperatively, the patient experienced recurrent nonsustained ventricular tachycardia episodes requiring prolonged monitoring without indication for implantable cardioverter-defibrillator placement. He was discharged on postoperative day 14, and both anginal symptoms and the episodes of ventricular tachycardia resolved during follow-up.

## Patient 5

**Figure 6:**
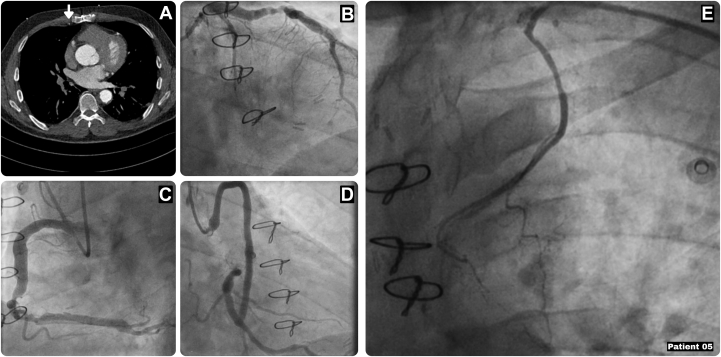
Characteristics of Patient 5 (A) Preoperative computed tomography displaying the RIMA still in situ (arrow). (B) Coronary angiography showing left main disease with severely ectatic native coronary arteries. (C) Coronary angiography showing the ectatic RCA. (D) Coronary angiography showing patent SVG-OM. (E) Coronary angiography showing a nonfunctioning LIMA-LAD graft. LAD = left anterior descending artery; LIMA = left internal mammary artery; OM = obtuse marginal branch; RCA = right coronary artery; RIMA = right internal mammary artery; SVG = saphenous vein graft.

A 58-year-old man with prior CABG presented with unstable angina (CCS class IV). His previous surgery included a LIMA-LAD and an aortic-SVG to the obtuse marginal branch (OM), and his medical history was notable for hypertension, hypercholesterolemia, heavy smoking, prostate cancer treated with prostatectomy, and prior thalamic infarction. Coronary angiography revealed left main disease and a nonfunctioning LIMA-LAD graft, with a patent SVG-OM and severely ectatic native coronary arteries (Figure 6). Echocardiography showed a dilated aortic root and ascending aorta (49 mm) with mild aortic regurgitation and preserved left ventricular function. A PCI was deemed unsuitable given severe coronary ectasia, and a redo Endo-CAB was therefore performed (EuroSCORE II: 3.16). The RIMA was harvested endoscopically from the left side, crossing the anterior mediastinum, and extended using a radial artery conduit. The postoperative course was uncomplicated, with discharge on postoperative day 4, complete resolution of angina, and successful smoking cessation at follow-up.

## Patient 6

**Figure 7:**
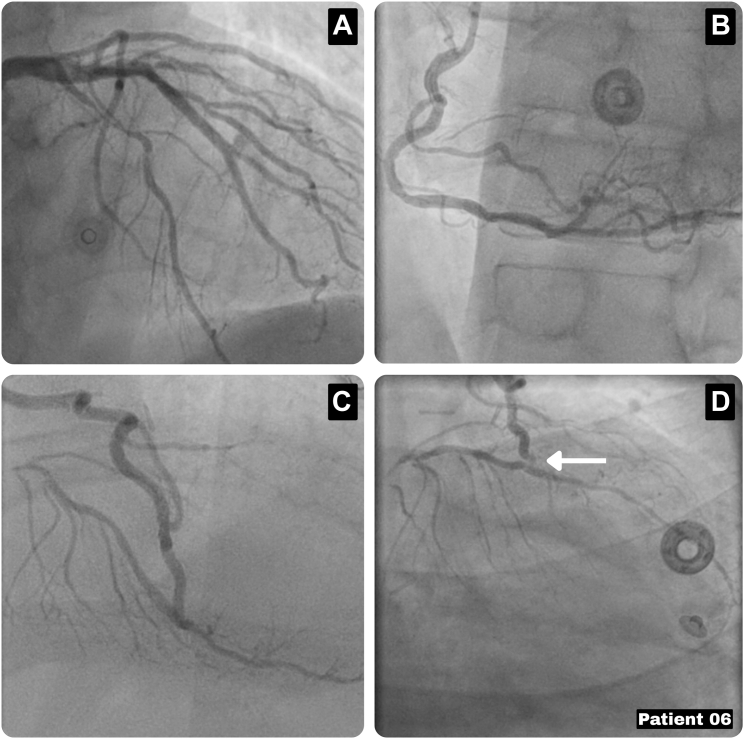
Characteristics of Patient 6 (A) Coronary angiography showing a long stenosis in the LAD and stenosis in the circumflex artery. (B) Coronary angiography showing the preoperative RCA. (C and D) Coronary angiography showing the LIMA-LAD, radial Y–IM graft in situ. Tapering is seen of the anastomosis of the LIMA-LAD graft (arrow). IM = intermediate branch; LAD = left anterior descending artery; LIMA = left internal mammary artery; RCA = right coronary artery.

A 55-year-old man with hypertension and prior left nephrectomy presented with recurrent angina (CCS class III). Initial evaluation revealed mildly reduced left ventricular function (left ventricular ejection fraction: 45%) and 2-vessel coronary artery disease, for which an Endo-CAB was performed with a LIMA-LAD graft and radial artery Y graft to the intermediate branch. The LIMA was harvested endoscopically, and the anastomosis was made through a mini-thoracotomy, as described above. Six months after the procedure, the patient re-presented with unstable angina, and coronary angiography showed the known native LAD stenosis with tapering of the LIMA-LAD graft resulting in anterior wall ischemia (Figure 7). A redo Endo-CAB was performed, with endoscopic adhesiolysis and identification of the LIMA and LAD coronary target. Afterward, the previous mini-thoracotomy was opened, and a radial artery was anastomosed on the proximal LIMA and the distal LAD. During test clamping of the LIMA, ventricular tachycardias occurred, for which the LIMA was opened again, and the procedure was performed under extracorporeal circulatory support. The recovery was uneventful, with discharge on postoperative day 5 and sustained symptom relief at follow-up.

## Discussion

This case series demonstrates that redo Endo-CAB is a feasible and effective revascularization strategy in patients with prior cardiac or thoracic surgery. Redo coronary revascularization is inherently challenging and is traditionally associated with increased morbidity and mortality when performed via repeat median sternotomy. An endoscopic-assisted, sternal-sparing approach offers a valuable alternative by significantly reducing surgical trauma while preserving the durability of surgical revascularization.

### Endoscopic IMA harvest and mini-thoracotomy during reoperations

Endoscopic IMA harvest during redo procedures has several advantages. It avoids a repeat median sternotomy, which carries a well-documented risk of injury to the heart, great vessels, or patent bypass grafts due to retrosternal adhesions. These risks are amplified in elderly patients and those with multiple comorbidities or prior IMA grafting.^8^ In patients with previous thoracic surgery, a sternotomy may in some cases constitute an untouched anatomical plane and should therefore be considered. However, a minimally invasive approach can also provide significant versatility by enabling bilateral IMA harvesting and thoracoscopic dissection of the ascending aorta, while avoiding re-entry through previously operated thoracic fields. The technique allows for conduit harvest on the contralateral side when prior thoracic interventions preclude ipsilateral access. The choice of approach should be individualized based on prior interventions, anatomical considerations, and the planned revascularization strategy.

Carbon dioxide insufflation and enhanced endoscopic visualization facilitate superior identification of target structures and coronaries, enabling intraoperative refinement of the revascularization strategy. In addition, the use of the needle-based localization method described by Ohtsuka et al^6^ allows for optimal placement of the mini-thoracotomy, further minimizing surgical trauma. The mini-thoracotomy is particularly advantageous in the reoperative setting, as it completely avoids sternal manipulation and is associated with reduced blood loss, shorter hospital stay, and faster recovery.^9^ Prior studies from our institution have demonstrated improved postoperative outcomes with Endo-CAB compared with conventional sternotomy, including shorter drain duration and earlier mobilization.^5^^,^^10^ These benefits are especially relevant in high-risk and elderly patients, in whom minimizing surgical stress is critical.

### PCI or redo CABG during coronary reintervention

The choice between PCI and surgical reintervention in patients with recurrent angina after prior CABG remains complex and highly individualized. PCI is often favored given its minimally invasive nature and lower short-term procedural risk, particularly in patients considered poor surgical candidates. However, in patients with unfavorable coronary anatomy or lesions unsuitable for PCI, surgical revascularization remains the only viable option.

Although redo CABG has been associated with superior long-term freedom from repeat revascularization, it carries an increased risk of perioperative mortality and complications.^2^ Redo Endo-CAB may help bridge this gap by combining the long-term durability of surgical revascularization with a substantially reduced procedural burden. In our series, we describe patients who required a graft to the LAD in a reoperative setting. For patients requiring multivessel grafting, a redo sternotomy procedure would still be the preferred option in our center. In carefully selected patients, a redo Endo-CAB may represent an optimal revascularization strategy for revascularization of the LAD.

**Visual Summary:**
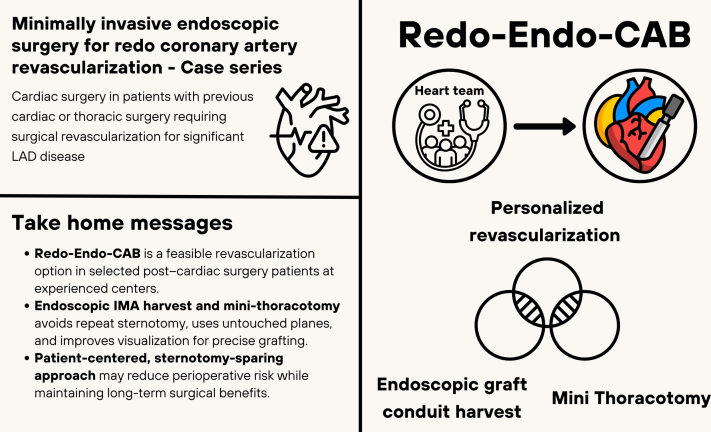
Redo-Endo-CAB Endo-CAB = endoscopic-assisted coronary artery bypass grafting; IMA = internal mammary artery; LAD = left anterior descending artery.

### Data Availability

Data will be made available by the corresponding author upon reasonable request.

## Funding Support and Author Disclosures

The authors have reported that they have no relationships relevant to the contents of this paper to disclose.
